# FDA Approval of Cardiac Valve Devices Implanted in a National Cohort of Pediatric Patients, 2016-2022

**DOI:** 10.1001/jamapediatrics.2025.0131

**Published:** 2025-03-24

**Authors:** Susmitha Wunnava, Timothy A. Miller, Meena Nathan, Florence T. Bourgeois

**Affiliations:** 1 Harvard-MIT Center for Regulatory Science, Harvard Medical School, Boston, Massachusetts; 2 Computational Health Informatics Program, Boston Children’s Hospital, Boston, Massachusetts; 3 Department of Pediatrics, Harvard Medical School, Boston, Massachusetts; 4 Department of Cardiac Surgery, Children’s Hospital, Boston, Massachusetts; 5 Department of Surgery, Harvard Medical School, Boston, Massachusetts

## Abstract

This cohort study evaluates the FDA approval status of cardiac valve devices implanted in pediatric patients.

Cardiac valve abnormalities are one of the most common congenital cardiac malformations in children; however, relatively few valve implant devices have been approved by the US Food and Drug Administration (FDA) for pediatric use. Therefore, off-label use of adult valves to treat valve disease in children is a standard practice.^1,2^ Challenges in developing pediatric-specific devices are largely related to barriers in performing clinical trials in small patient populations and perceived low market potential by manufacturers.^3^ To define gaps in treatment options and support efforts to advance pediatric device research and development, we analyzed data from a large database of cardiac operations performed in the US and evaluated the FDA approval status of cardiac valve devices implanted in pediatric patients.

## Methods

Data were obtained from the Society of Thoracic Surgeons Congenital Heart Surgery Database, which collects information on operations for congenital and acquired heart defects in children across 120 surgical centers in North America (eMethods in Supplement 1).^4^ This study cohort consisted of valve implant procedures performed in the US from January 1, 2016, to June 30, 2022, for patients younger than 18 years. Information on race and ethnicity was not available. This research was determined to be exempt research with a waiver of informed consent from the Advarra Institutional Review Board because it used nonidentifiable patient data. The study followed the STROBE reporting guideline.

For each procedure, all implanted valve devices were identified and device information retrieved from FDA medical device databases.^5^ Devices were categorized as FDA approved for pediatric use, FDA approved for adults only, FDA approved with unknown pediatric approval status, and not FDA approved for any patient population. We also identified all autografts and surgeon-fashioned valves, which do not fall under FDA regulation. We used descriptive statistics to characterize valve implant procedures and FDA approval status of valve implant devices.

## Results

The study cohort consisted of 9608 valve implant procedures for 8971 pediatric patients (median age, 9.0 [IQR, 2.7-14.0] years). The primary diagnosis and corresponding surgical procedures varied by age, with the most common procedures consisting of Norwood procedures for neonates (161 [35.2%]), mitral valve replacement for infants (258 [15.8%]), and pulmonic valve replacement/conduit operation for children (1203 [30.1%]) and adolescents (1127 [32.1%]) (Table 1).

**Table 1.** Patient and Clinical Characteristics of Pediatric Valve Device Implant Procedures

Characteristic	No. (%)^a^				
	All procedures (N = 9608)	Neonates (n = 458)	Infants (n = 1636)	Children (n = 4003)	Adolescents (n = 3511)
**Patient characteristics**					
Sex					
Male	5660 (58.9)	266 (58.1)	872 (53.3)	2297 (57.4)	2225 (63.4)
Female	3863 (40.2)	189 (41.3)	747 (45.7)	1673 (41.8)	1254 (35.7)
Other^b^	85 (0.9)	3 (0.7)	17 (1.0)	33 (0.8)	32 (0.9)
Age, median (IQR)	9.0 y (2.7-14.0)	7.0 d (4.0-12.0)	6.0 mo (3.8-10.5)	7.2 y (4.6-9.8)	15.0 y (13.5-16.5)
**Clinical and operative characteristics**					
Most common indications for surgical intervention^c^					
Pulmonary valve disease or conduit failure	3285 (34.2)	6 (1.3)	241 (14.7)	1639 (40.9)	1399 (39.8)
Aortic valve disease	1634 (17.0)	50 (10.9)	213 (13.0)	564 (14.1)	807 (23.0)
Mitral valve disease	1110 (11.6)	13 (2.8)	320 (19.6)	470 (11.7)	307 (8.7)
Truncus arteriosus	323 (3.4)	91 (19.9)	54 (3.3)	107 (2.7)	71 (2.0)
Hypoplastic left heart syndrome	198 (2.1)	133 (29.0)	31 (1.9)	29 (0.7)	5 (0.1)
Most common surgical procedures performed^d^					
Pulmonic valve replacement or conduit operation	2610 (27.2)	23 (5.0)	257 (15.7)	1203 (30.1)	1127 (32.1)
Mitral valve replacement	942 (9.8)	5 (1.1)	258 (15.8)	420 (10.5)	259 (7.4)
Pulmonary artery plasty	852 (8.9)	7 (1.5)	123 (7.5)	482 (12.0)	240 (6.8)
Right ventricular outflow tract procedure	566 (5.9)	0 (0.0)	35 (2.1)	256 (6.4)	275 (7.8)
Ross-Konno procedure	326 (3.4)	28 (6.1)	123 (7.5)	121 (3.0)	54 (1.5)
Norwood procedure^e^	176 (1.8)	161 (35.2)	15 (0.9)	0 (0.0)	0 (0.0)
Truncus arteriosus repair	86 (0.9)	72 (15.7)	12 (0.7)	2 (0.0)	0 (0.0)
Yasui procedure	60 (0.6)	34 (7.4)	24 (1.5)	2 (0.0)	0 (0.0)
No. of device implants per procedure					
1	8384 (87.3)	302 (65.9)	1447 (88.4)	3565 (89.1)	3070 (87.4)
≥2	1224 (12.7)	156 (34.1)	189 (11.6)	438 (10.9)	441 (12.6)

^a^ Pediatric age groups were defined as neonates (aged 0-28 days), infants (aged 29 days to <2 years), children (aged 2 to <12 years), and adolescents (aged 12-17 years).

^b^ Other includes ambiguous and unspecified gender categories.

^c^ All surgical interventions with the 3 highest frequencies within each age group.

^d^ All surgical procedures with the 3 highest frequencies within each age group.

^e^ Norwood procedures were all right ventricle to pulmonary artery conduits.

The 9608 valve procedures involved implantation of 10 875 valve devices, of which 965 (8.9%) were FDA approved for pediatric use, whereas 6716 (61.8%) were FDA approved for adults only (Table 2). Additionally, 736 (6.8%) devices were FDA approved with unknown pediatric approval status, 1076 (9.9%) were not FDA approved, and 392 (3.6%) could not have their FDA approval ascertained. The devices used in these procedures corresponded to a total of 186 unique brand name devices, of which 8 (4.3%) were approved for children, 150 (80.6%) approved for adults only, 14 (7.5%) approved for adults with unknown pediatric approval status, and 14 (7.5%) not FDA approved for any patient population.

**Table 2.** FDA Approval Status of Valve Devices by Age Group and Implant Location

Variable	Implanted devices, No.	No. (%)						
		FDA approved for pediatric use	FDA approved for adults only	FDA approved with unknown pediatric approval status	Not FDA approved	Unknown FDA approval status	Autograft	Surgeon- fashioned
Total	10 875	965 (8.9)	6716 (61.8)	736 (6.8)	1076 (9.9)	392 (3.6)	590 (5.4)	400 (3.7)
Age group^a^								
Neonate	624	33 (5.3)	180 (28.8)	229 (36.7)	77 (12.3)	14 (2.2)	24 (3.8)	67 (10.7)
Infant	1830	379 (20.7)	743 (40.6)	134 (7.3)	263 (14.4)	61 (3.3)	109 (6.0)	141 (7.7)
Child	4453	436 (9.8)	2794 (62.7)	208 (4.7)	479 (10.8)	170 (3.8)	234 (5.3)	132 (3.0)
Adolescent	3968	117 (2.9)	2999 (75.6)	165 (4.2)	257 (6.5)	147 (3.7)	223 (5.6)	60 (1.5)
Implant location								
Pulmonic valve	6905	795 (11.5)	4129 (59.8)	496 (7.2)	226 (3.3)	974 (14.1)	21 (0.3)	264 (3.8)
Aortic valve	1810	13 (0.7)	999 (55.2)	63 (3.5)	58 (3.2)	76 (4.2)	565 (31.2)	36 (2.0)
Mitral valve	1379	134 (9.7)	1058 (76.7)	56 (4.1)	69 (5.0)	12 (0.9)	3 (0.2)	47 (3.4)
Tricuspid valve	592	13 (2.2)	396 (66.9)	98 (16.6)	31 (5.2)	6 (1.0)	0	48 (8.1)
Truncal valve	94	4 (4.3)	74 (78.7)	4 (4.3)	7 (7.4)	4 (4.3)	0	1 (1.1)
Common atrioventricular valve	72	6 (8.3)	51 (70.8)	6 (8.3)	1 (1.4)	4 (5.6)	1 (1.4)	3 (4.2)
Unspecified	23	0	9 (39.1)	13 (56.5)	0	0	0	1 (4.3)

Abbreviation: FDA, US Food and Drug Administration.

^a^ Pediatric age groups were defined as neonates (aged 0-28 days), infants (aged 29 days to 2 years), children (aged 2 to <12 years), and adolescents (aged 12-17 years).

Use of approved pediatric valves was highest among infants (379 [20.7%]), followed by children (346 [9.8%]), neonates (33 [5.3%]), and adolescents (117 [2.9%]). The most common locations for use of an approved pediatric valve device were the pulmonic and mitral valve locations, with 795 (11.5%) and 134 (9.7%) devices, respectively, approved for pediatric use.

## Discussion

In this national cohort study of pediatric patients undergoing valve implantation surgery, less than 10% of implanted devices were FDA approved for pediatric patients, with most procedures involving off-label use of adult devices. A limitation is that we were unable to account for device implantation in nonapproved locations, potentially underestimating the extent of off-label device use.

Previous work quantifying off-label drug use in children has been instrumental in informing research priorities and guiding regulatory programs,^6^ but such work remains limited for devices. Our findings provide information on current practices in the treatment of children with cardiac valve disease and indicate that additional strategies are needed to increase pediatric-specific development and approval of valve implant devices.

## Notes

Supplement 1.
eMethods.

Supplement 2.
Data Sharing Statement
