# Supplementary material for: FDA Approval of Cardiac Valve Devices Implanted in a National Cohort of Pediatric Patients, 2016-2022
Source: JAMA Pediatr. 2025 Mar 24;179(5):570–3. doi: 10.1001/jamapediatrics.2025.0131 (PMC11933990; doi:10.1001/jamapediatrics.2025.0131)
Supplement: Supplement 1. — eMethods. [file jamapediatr-e250131-s001.pdf]

Supplemental Online Content

Wunnava S, Miller TA, Nathan M, Bourgeois FT. FDA approval of cardiac valve devices implanted in a national cohort of pediatric patients, 2016-2022. *JAMA Pediatrics*. Published online March 24, 2025. doi:10.1001/jamapediatrics.2025.0131

eMethods. Supplemental Methods

This supplemental material has been provided by the authors to give readers additional information about their work.

© 2025 Wuvvana S et al. *JAMA Pediatrics*.

eMethods. Supplemental Methods

Data Source

The Society of Thoracic Surgeons Congenital Heart Surgery Database captures over 39,000 surgeries per year, with trained abstractors using standardized forms to collect data on demographic, clinical, and procedural features for all operative encounters at participating centers. After extensive data verification and processing, information is available on preoperative, intraoperative, and early postoperative characteristics, including patients' underlying cardiac diagnosis, primary reason for surgical intervention, details of the procedures performed, devices implanted, clinical outcomes, and disposition at the end of an episode of care.

Classification of Valve Implant Devices

Valve implant devices captured in the database include mechanical valves, bioprosthetic valves, allograft valves, valve conduits, annuloplasty bands and rings, autografts, and surgeon-fashioned valves. Device labels were reviewed to determine whether the devices were approved for pediatric (<18 years) use. Labels for original as well as supplemental approvals were considered to capture pediatric approvals added after initial market introduction. The pediatric approval status of a device was defined based on approval in pediatric patients and did not account for device implantation in anatomic locations other than the approved locations. Devices that were FDA approved but did not include information on pediatric approval were classified as FDA approved with unknown pediatric approval status. In addition, devices that could not be uniquely identified in the FDA databases because the device name was missing, were classified as having an unknown FDA approval status.

© 2025 Wuvvana S et al. *JAMA Pediatrics*.
