# Supplementary material for: FDA Approval of Cardiac Valve Devices Implanted in a National Cohort of Pediatric Patients, 2016-2022
Source: JAMA Pediatr. 2025 Mar 24;179(5):570–3. doi: 10.1001/jamapediatrics.2025.0131 (PMC11933990; doi:10.1001/jamapediatrics.2025.0131)
Supplement: Supplement 2. — Data Sharing Statement [file jamapediatr-e250131-s002.pdf]

Data Sharing Statement

Wunnava. FDA Approval of Cardiac Valve Devices Implanted in a National Cohort of Pediatric Patients, 2016-2022. *JAMA Pediatr*. Published March 24, 2025.
doi:10.1001/jamapediatrics.2025.0131

Data
Data available: No

Additional Information
Explanation for why data not available: The data are available from the Society of Thoracic Surgeons upon request. Our data use agreement does not allow direct sharing of the registry data with investigators.
